# A comprehensive analysis of meloxicam particles produced by nanosecond laser ablation as a wet milling technique

**DOI:** 10.1038/s41598-022-16728-9

**Published:** 2022-07-22

**Authors:** Eszter Nagy, Zsolt Homik, Tamás Smausz, Judit Kopniczky, Máté Náfrádi, Tünde Alapi, David Kokai, Katalin Burián, Piroska Szabó-Révész, Rita Ambrus, Béla Hopp

**Affiliations:** 1 Department of Optics and Quantum Electronics, University of Szeged, Dóm tér 9, Szeged, 6720 Hungary; 2 Department of Inorganic and Analytical Chemistry, University of Szeged, Dóm tér 7, Szeged, 6720 Hungary; 3 Department of Medical Microbiology, Albert Szent-Györgyi Medical School, University of Szeged, Dóm tér 10, Szeged, 6720 Hungary; 4 Institute of Pharmaceutical Technology and Regulatory Affairs, University of Szeged, Eötvös utca 6, Szeged, 6720 Hungary

**Keywords:** Materials science, Optics and photonics, Physics, Drug discovery, Pharmaceutics

## Abstract

Recently, the number of water insoluble and poorly soluble drug compounds has increased significantly. Therefore, growing interest has been witnessed in different particle size reduction techniques to improve the dissolution rates, transport characteristics and bioavailability of drugs. Laser ablation has proven to be an alternative method to the production of nano- and micrometre-sized drug particles without considerable chemical damage. We present the nanosecond laser ablation of drug pastilles in distilled water, targeting meloxicam, a poorly water soluble nonsteroidal anti-inflammatory drug, at different laser wavelengths (248 nm, 532 nm and 1064 nm). Besides chemical characterization, crystallinity, morphology and particle size studies, the mechanism of the particle generation process was examined. The applicability of ablated particles in drug formulation was investigated by solubility, cytotoxicity and anti-inflammatory effect measurements. We showed that laser ablation is a clean, efficient and chemically non-damaging method to reduce the size of meloxicam particles to the sub-micrometre–few micrometre size range, which is optimal for pulmonary drug delivery. Complemented by the excellent solubility (four to nine times higher) and anti-inflammatory (four to five times better) properties of the particles compared to the initial drug, laser ablation is predicted to have wider applications in the development of drug formulations.

## Introduction

Solubility is an increasing concern for the pharmaceutical industry as poorly soluble drugs represent around 40% of the marketed and 90% of the candidate compounds^1^. This tendency inspired the development of various drug formulation and administration strategies^2^. One of the simplest physical methods to increase solubility and bioavailability is particle size reduction^3,4^, the techniques of which can be classified^5^ as a) conventional: milling^6,7^, spray drying^8^, high-pressure homogenization^7^ or b) non-conventional: liquid anti-solvent crystallization^9^, spray freeze-drying^10^, supercritical fluid-based micronization processes^11^ and pulsed laser ablation^12–15^.

Pulsed laser ablation is a flexible material processing technique that can be used in vacuum, gas or liquid with diverse target materials. It is widely applied to synthesize different nanomaterials^16–19^. Since laser ablation is a chemical-free technique that can be precisely controlled over a broad range of parameters, it has also become attractive for life sciences. It was shown that drug particles could be shredded by laser ablation, thus reducing the size of the original (synthesized) particles down to the nanometre–micrometre size range^12–15^.

The present work complements our previous investigation of meloxicam suspension production using pulsed laser ablation in liquid (PLAL)^12^. Three different laser wavelengths were used, and besides the structure and composition of the ablated particles, their solubility, cytotoxicity and anti-inflammatory activity were studied and compared to the original drug. To understand the underlying process, we tracked the ablation of the drug pastilles by fast photography.

## Materials and methods

### Meloxicam & targets

Meloxicam (Mx.) (4-hydroxy-2-methyl-N-(5-methyl-2-thiazolyl)2H-benzothiazine-3-car-boxamide-1,1-dioxide) was obtained from EGIS Ltd., (Budapest, Hungary) in 100% crystalline powder form with a pharmaceutical grade above 99% and a median particle size of d(0.5) = 27.52 µm.

Pure meloxicam targets were produced by compressing 300 mg meloxicam powder into pastilles with a diameter of 9 mm using 15 kN compacting force in a KORSCH EK-0 Tablet Press machine (KORSCH AG—Berlin, Germany).

### Laser sources & setups

A KrF excimer laser (LLG Twinamp, λ = 248 nm, FWHM = 18 ns, f = 10 Hz) and a frequency doubled Q-switched Nd:YAG laser (Quantel, λ = 532 nm/1064 nm, FWHM = 6 ns, f = 10 Hz) provided the nanosecond pulses at three wavelengths from the ultraviolet through the visible to the infrared region.

**Figure 1:**
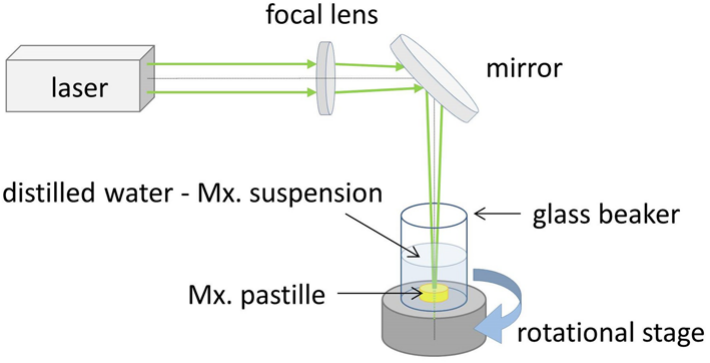
Experimental setup for pulsed laser ablation in liquid (PLAL).

The setup was the same for all three wavelengths (Fig. 1): the laser beam was transmitted through a fused silica lens and focused just below the surface of the target placed in a rotating glass beaker containing 10 ml distilled water. The target surface was approximately 5 mm below the water level. The spot size was ∼0.6 mm^2^ and the pulse energy was ∼60 mJ at λ = 532 nm/1064 nm, and ∼0.3 mm^2^ and ∼30 mJ at λ = 248 nm, respectively. The ablation fluence was 9.4 J/cm^2^ in all cases, and depending on the amount of ablated particles required for a particular measurement, approximately 36,000 or 72,000 laser pulses were used. After ablation, the resulting suspension was filtered through a sieve (300 µm pore size) to remove flaked pieces of the pastilles so that they would not distort the analysis of the laser ablated particles.

### Fourier transform infrared spectroscopy (FTIR) measurements

The water content of the suspension was evaporated at 50 °C in a laboratory oven (CARBOLITE 2416, Thermal Engineering Services Ltd., Worcester, England) within 12 h, and the remaining powder was used for FTIR sample preparation. A few mg of the dried particles were mixed with 150 mg of KBr, and this mixture was ground in an achate mortar and pressed into a disk with a diameter of 13 mm at 10 kN for FTIR analysis.

FTIR spectra were recorded with an AVATAR 330 FTIR spectrometer (Thermo Nicolet, LabX Midland, ON, Canada) between 4000 and 400 cm^−1^, with 4 cm^−1^ resolution and averaging 128 scans/measurement using baseline correction.

### Raman spectroscopy measurements

For Raman spectroscopy studies, a small amount of the same dried powder was used as for the FTIR measurements. The Thermo Scientific™ DXR™ Raman microscope (Thermo Fisher Scientific Inc., Waltham, MA, USA) was operated at λ = 780 nm laser wavelength for excitation at 2 mW laser power and with an approximately 3.1 µm spot size. Raman spectra were recorded in the 2500 and 500 cm^−1^ wavenumber range using a 400 lines/mm grating. The resolution was 4.7–8.7 cm^−1^. All spectra were recorded 20 times with a 2 s integration time.

### High performance liquid chromatography and mass spectrometry (HPLC–MS) studies

HPLC–MS was used for the separation and identification of the ablation products. After ablation, the resulting suspension was concentrated to 1–2 ml by evaporation in a laboratory oven at 50 °C. Then, the solid particles were dissolved by adding 5 ml acetonitrile. Before the HPLC–MS analysis, each sample was filtered through a syringe filter (mean pore diameter: 0.22 µm).

The Agilent 1100 HPLC system was equipped with a diode array detector (DAD) and an Agilent LC/MSD/VL mass spectrometer (MS) (Agilent Technologies, Palo Alto, CA, USA). The separation was performed using a Kinetex 2,6u XB-C18 100A (Phenomenex) column, thermostated at 35 °C. The eluent consisted of 40 v/v% methanol and 60 v/v% water with formic acid (0.1 v/v%) (flow rate 0.80 ml min^-1^). The detection wavelengths were 210 nm and 350 nm. The MS measurements were performed using electrospray ionization (ESI) in positive ion mode, 300 °C nitrogen drying gas, 3500 V capillary voltage and 50 V fragmentor voltage were applied. Meloxicam concentration of the suspensions was determined by HPLC–MS, using the calibration curve. The first-order calibration curve (R^2^ = 0.991) was determined with pure meloxicam solutions in the range of 50–500 mg/dm^3^.

### X-ray powder diffractometer (XRPD) investigations

For crystallinity studies, we used the powder obtained from the suspension after it was dried at 50 °C for 12 h. We employed a BRUKER D8 Advance X-ray powder diffractometer (Bruker AXS GmbH, Karlsruhe, Germany) with Cu K λI radiation (λ = 1.5406 Å) and a VÅNTEC-1 detector, scanning the samples at 40 kV and 40 mA, with a 2θ angular range of 3° to 40°, at a scan speed of 0.1 s/step and a step size of 0.0074°.

### Scanning electron microscopy (SEM) studies

Morphology and particle size were studied by scanning electron microscopy (SEM). After ablation, a drop of the stirred suspension was placed on a silicon plate. Prior to imaging, the droplet was dried and then coated with gold using a sputter-coater (Bio-Rad SC 502, VG Microtech, Uckfield, UK). The SEM studies were performed with a Hitachi S-4700 SEM system (Hitachi S4700, Hitachi Scientific Ltd., Tokyo, Japan).

### Fast photography studies

**Figure 2:**
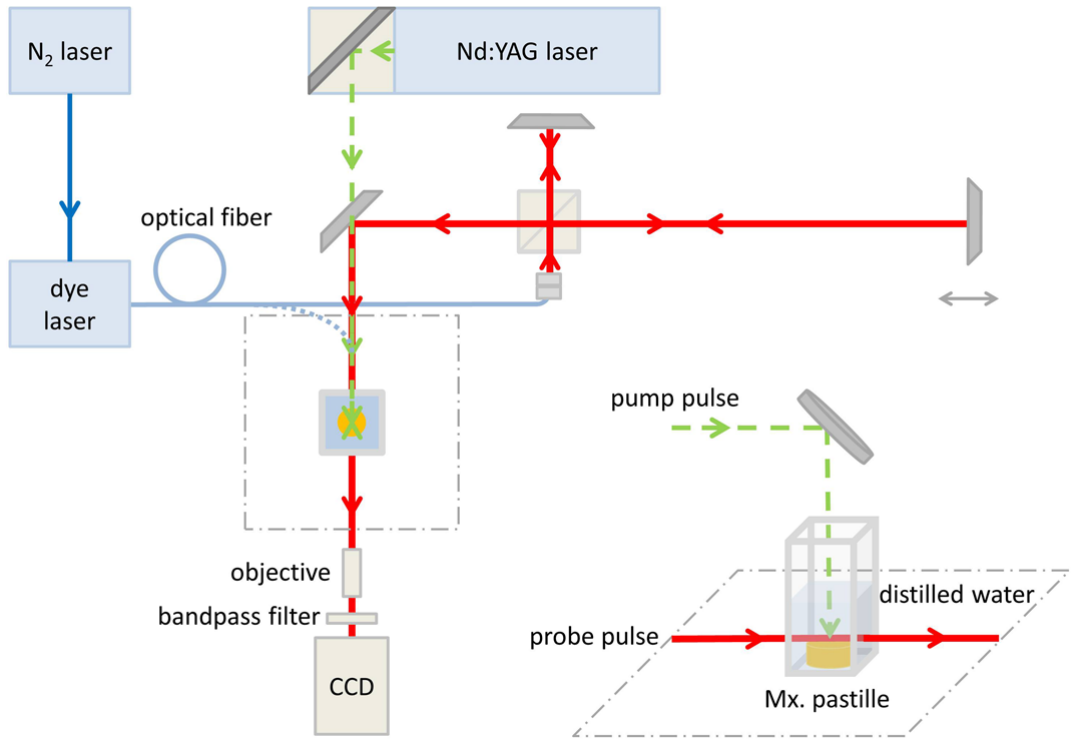
Pump-probe setup for fast photography with direct probe illumination (blue dotted line) or extended with a Michelson interferometer for double exposition with two probe pulses.

To describe the particle generation process, we built a pump-probe system suitable for fast photography (Fig. 2).

The SEM images implied that the particle fabrication process was independent of the ablation wavelength. For the investigations, a Nd:YAG laser (Quantel, λ = 532 nm/1064 nm, FWHM = 6 ns) was chosen as a pump (ablating) source. At λ = 532 nm, the spot size was ∼0.2–0.3 mm^2^ and the pulse energies were ∼6–8 mJ and ∼12–16 mJ, resulting in F = 3 J/cm^2^ and F = 6 J/cm^2^ fluences, respectively. At λ = 1064 nm, the spot size was ∼0.07–0.09 mm^2^ and the pulse energy was ∼15 mJ, resulting in F = 18 J/cm^2^ fluence. (The spot size was reduced compared to the sample generation experiments in order to fit into the field of view.) As probe beam, a nitrogen laser induced Rhodamine 6G dye laser was used (λ = 590 nm, FWHM = 1 ns). The probe laser beam was transported to the ablation setup by an optical fibre and forwarded either to the target directly or through a Michelson interferometer which provided two consecutive probe pulses with 8 ns time separation. The direct illumination was used to investigate the temporal evolution of particle generation, while the purpose of using two probe pulses was to study the initial, transient part of the ablation process. The time delay between the pump and the first probe pulse was monitored using a photodiode. The pump pulses hit the target from above, while the probe pulses provided illumination from the side (tangential to the illuminated surface), projecting a shadow picture of the ablation process on a CCD camera (The Imaging Source-DMK 23G445, 30 fps, N = 3 ×). The light of the generated plasma was filtered out by a bandpass optical filter placed in front of the camera. The separate triggering of the pump and the probe lasers and the CCD camera with delays in the nanosecond to millisecond range was controlled by a digital delay generator (DDG) (Stanford Research Systems-DG645).

### Solubility measurements

Solubility measurements were carried out at room temperature (22 °C), over a time span of 24 h, in 10 ml phosphate buffer solution (pH = 7.4), using an AvaSpec-2048L probe and an AvaLight DH-S-BAL spectrophotometer (Avantes, Apeldoorn, The Netherlands). Each sample solution was filtered (filter mean pore diameter: 0.45 µm), and after the required dilution, the absorbance was measured at λ = 362 nm and the meloxicam concentration was calculated.

### Cytotoxicity measurements

After the suspensions were dried (50 °C, 12 h), 1 mg of the solid residue was re-suspended in 1 ml minimum essential medium Eagle with Earle's salts (Sigma, St Louis, MO, USA), supplemented with 10% vol/vol foetal calf serum, 0.5% wt/vol glucose, 0.3 mg of l-glutamine ml^−1^, 4 mM HEPES and 25 µg of gentamycin ml^−1^.

To characterize cell viability, the mitochondrial activity was measured using an MTT (3-(4,5-dimethylthiazol-2-yl)-2,5-diphenyltetrazolium bromide) assay. During these experiments, A549 (adenocarcinomic human alveolar basal epithelial cells) (ATCC) were used and seeded at a density of 4∙10^4^ cells/well. The cells were treated with a sample containing particles produced by laser ablation of meloxicam pastilles at 248, 532 or 1064 nm wavelengths. Two-fold serial dilution of compounds has been prepared with a maximum compound concentration of 1 mg/ml, and cytotoxicity was determined with 24 h incubation time at 37 °C. As the next step, 20 μl of thiazolyl blue tetrazolium bromide (MTT; Sigma, St. Louis, Missouri, USA) was added to each well. Following additional incubation for 4 h at 37 °C, sodium dodecyl sulphate (Sigma, St. Louis, Missouri, USA) solution (10% in 0.01 M HCI) was added and incubated overnight. Finally, cytotoxicity was determined by optical density (OD) measurements at 550 nm (reference: 630 nm) using an EZ READ 400 ELISA reader (Biochrom, Cambridge, United Kingdom). At each concentration the assay was repeated four times. Cell viability was concluded based on the following formula: ${1}00 - \left( {\left( {{\text{OD}}_{{{\text{sample}}}} - {\text{OD}}_{{{\text{medium}}\;{\text{ control}}}} } \right) / \left( {{\text{OD}}_{{{\text{cell }}\;{\text{control}}}} - {\text{OD}}_{{{\text{medium}}\;{\text{ control}}}} } \right)} \right) \times {1}00$ .

### Anti-inflammatory effect measurements

To observe the anti-inflammatory effect, A549 cells were seeded in 6-well plates at a density of 1∙10^6^ cells/well and treated with a mixture of 5 µg/ml lipopolysaccharide (LPS; ThermoFisher Scientific Waltham, MA, USA) and the solutions of the ablated particles or treated with 5 µg/ml LPS (positive control) or left untreated. The particle concentration in the solutions was chosen to be the maximum non-toxic concentration, which has been determined with the cytotoxicity measurements described in the previous section. The non-toxic concentrations were measured as 0.125 mg/ml, 0.016 mg/ml and 0.125 mg/ml for particles produced by ablation at 248 nm, 532 nm and 1064 nm, respectively.

#### Total RNA extraction and cDNA synthesis

The RNA was extracted after 24 h of treatment utilizing TRI reagent (Sigma-Aldrich, St. Louis, Missouri, USA) according to the manufacturer’s instructions. After that, 2 µg of RNA was reverse transcribed using Maxima Reverse Transcriptase in accordance with the manufacturer’s protocol, applying Random Hexamer primers (Thermo Fisher Scientific, Waltham, Massachusetts, USA).

#### qPCR amplification of IL-6, Actb

For qPCR a Bio-Rad CFX96 real-time system was used with the 5 × HOT FIREPol® EvaGreen® qPCR Supermix (Solis BioDyne, Tartu, Estonia) and the listed human-specific primer pairs: IL-6: 5’-CAGCTATGAACTCCTTCTCCAC-3’, and 5’-GCGGCTACATCTTTGGAATCT -3’; Actb: 5’-TTCTACAATGAGCTGCGTGTGGCT-3’, and 5’-TAGCACAGCCTGGATAGCAACGTA -3’ Primers were designed with the Primer Quest Tool software and synthesized by Integrated DNA Technologies Inc. (Montreal, Quebec, Canada). To verify the amplification specificity, a melting curve analysis was performed. Threshold cycles (Ct) were determined for IL-6 and Actb, and the relative gene expression was calculated with the 2-(ΔΔCt) method. One-way analysis of variance with repeated measurements (ANOVA RM) and planned comparisons were applied to compare statistical differences in log2(ΔΔCt) values between treated and control samples, as detailed previously, with a level of significance of *P* < *0.05*^20^.

#### Enzyme-linked immunosorbent assay (ELISA)

Following treatment for 24 h, the supernatant of the cells was collected and the IL-6 concentration was determined using standard sandwich human IL-6 ELISA kits Legend Max™ (BioLegend, San Diego, California, USA) in accordance with the manufacturer's instructions. The supernatant of LPS-treated cells was diluted 10 × . The kit had a dynamic range between 7.8 and 500 pg/ml. A Biochrom Anthos 2010 microplate reader (Biochrom, Cambridge, United Kingdom) was used to analyse the plates. Samples were assayed in duplicate.

## Results and discussion

### Chemical analysis (FTIR, Raman, HPLC)

FTIR, Raman and HPLC–MS studies were performed to determine the chemical composition of the particles produced by laser ablation.

**Figure 3:**
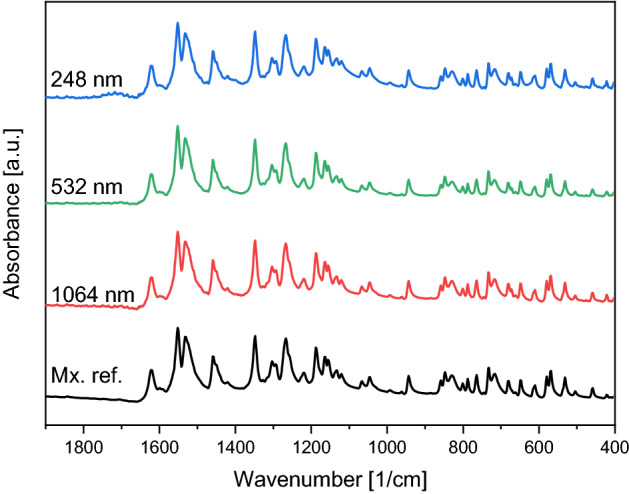
Fingerprint region of the FTIR spectra of the particles prepared by PLAL at different wavelengths (248 nm, 532 nm, 1064 nm) and the meloxicam (Mx. ref.). All spectra were normalized to the peak at ~ 1550 cm^−1^.

By comparing the fingerprint regions (1800–400 cm^−1^), we found that the FTIR spectra of the ablated particles were identical to the reference meloxicam spectrum (Mx. ref.) for all ablation laser wavelengths (Fig. 3): the characteristic peaks appeared at the expected wavenumbers and the peak intensity ratios were the same.

**Figure 4:**
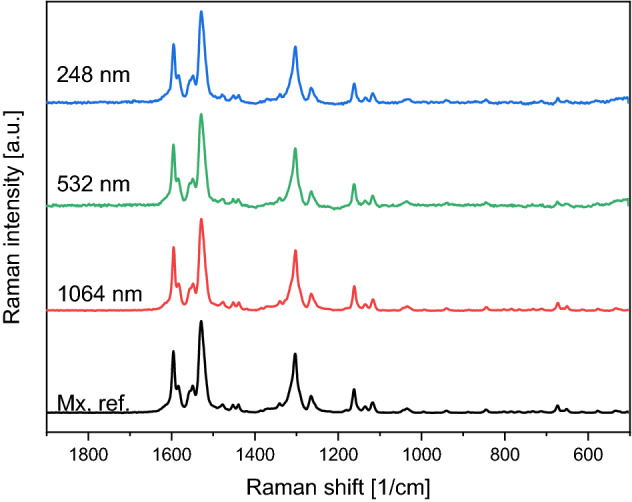
Raman spectra of the particles prepared by PLAL at different wavelengths (248 nm, 532 nm, 1064 nm) and the meloxicam (Mx. ref.). All spectra were normalized to the peak at ~ 1530 cm^−1^.

Similarly, the Raman spectra of the PLAL generated particles matched the spectrum of the initial meloxicam powder (Mx. ref.) for all wavelengths applied (Fig. 4). Thus, the Raman results agreed with the FTIR results.

We also analysed the ablated material by HPLC–MS to identify the products and their relative quantities.

**Figure 5:**
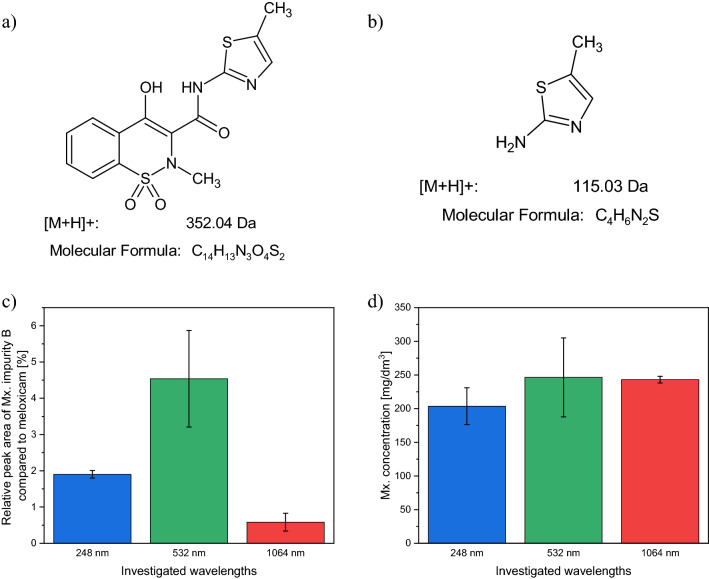
The chemical structure and ion mass of **(a)** meloxicam and **(b)** meloxicam impurity B. **(c)** The relative peak areas of meloxicam impurity B compared to meloxicam [(Area_Impurity_/Area_Meloxicam_) × 100%] and **(d)** the meloxicam concentrations (determined by HPLC–MS method) in case of the investigated wavelengths (248 nm, 532 nm, 1064 nm).

At all wavelengths, most of the material produced by ablation was meloxicam. In the mass spectra, besides meloxicam (C_14_H_13_N_3_O_4_S_2_, Fig. 5a) we found the product C_4_H_6_N_2_S (Fig. 5b), which is known as meloxicam impurity B^21–23^. Meloxicam impurity B is a section of the meloxicam molecule and is classified as pharmaceutical primary standard^24,25^. For all three ablation wavelengths, the peak area of Mx. impurity B was less than 5% of the meloxicam peak area (Fig. 5c). In addition, at least three, presumably aromatic products were present with relatively small peak areas.

After dissolving all the particles in the suspensions obtained by ~ 36,000 laser pulses at each wavelength, the meloxicam concentration was measured to be 203.56 ± 27.30, 246.32 ± 58.57 and 242.92 ± 4.90 mg/dm^3^ for 248 nm, 532 nm and 1064 nm laser wavelengths, respectively (Fig. 5d). These concentrations show that the yield of meloxicam is of the same order of magnitude at all applied wavelengths.

### Morphology and size (XRPD, SEM)

**Figure 6:**
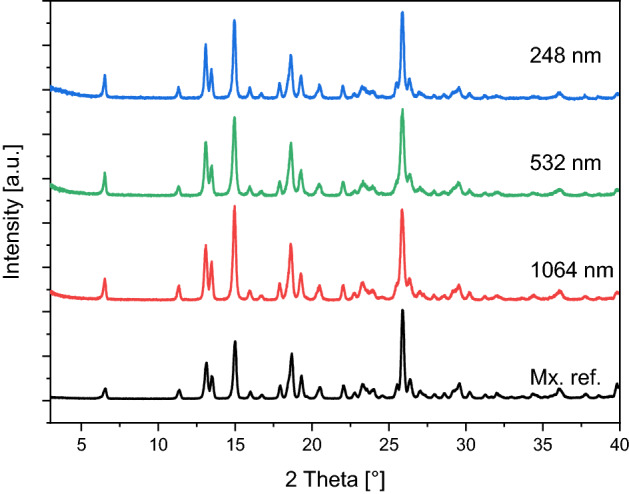
XRPD spectra of the particles prepared by PLAL at different wavelengths (248 nm, 532 nm, 1064 nm) and the meloxicam (Mx. ref.). Spectra were normalized to the peak at 25.94°.

We analysed the crystallinity of the produced particles by X-ray diffraction spectroscopy (Fig. 6). The XRPD pattern of our samples matched the spectra of the original meloxicam powder (Mx. ref.) indicating that the ablation did not change the crystalline structure. This proved that the laser ablation generated particles had approximately the same crystallinity index as the marketed meloxicam powder.

**Figure 7:**
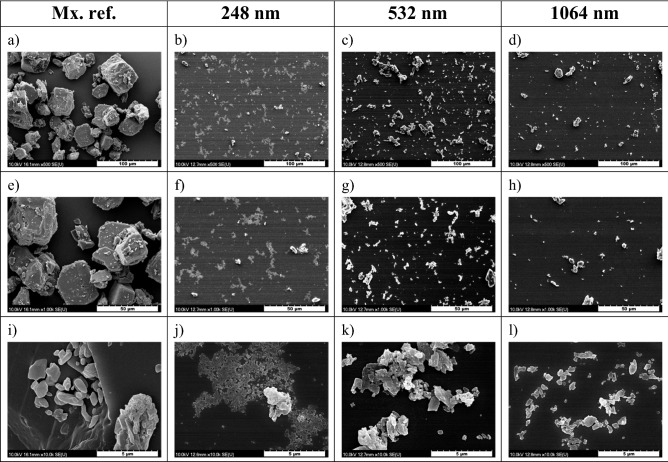
SEM images of the commercially available meloxicam powder (Mx. ref.) and the particles generated by PLAL at 248 nm, 532 nm and 1064 nm. Magnification: **(a)–(d)**: 500; **(e)–(h)**: 1 k; **(i)–(l)**: 10 k.

Morphology and particle size were investigated by SEM, and a few typical images are shown in Fig. 7. The PLAL-generated particles resembled shredded crystal pieces, indicating that mechanical fragmentation might be the relevant size reduction effect. In case of UV ablation (λ = 248 nm), islands of recrystallized material formed a film-like structure on the silicon plate.

**Figure 8:**
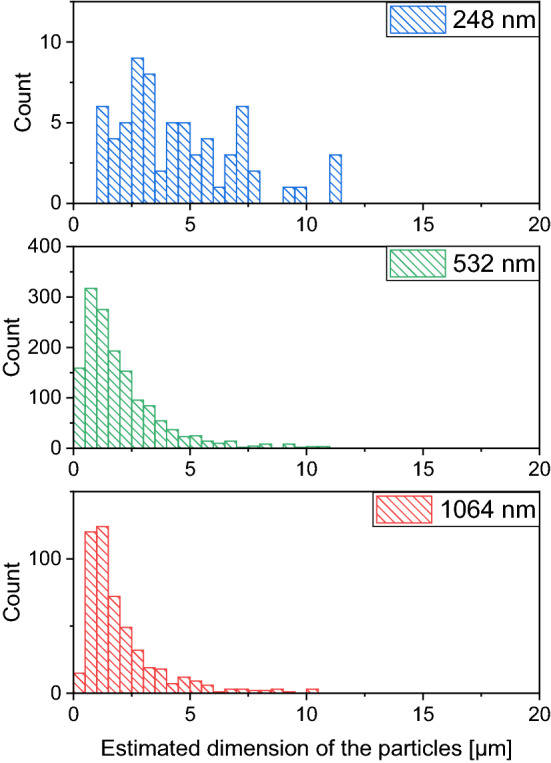
Size distribution of the particles generated by laser ablation at 248 nm, 532 nm and 1064 nm wavelengths. (Note the different scales on the y-axis).

Compared with the commercially available meloxicam powder, significant size reduction was achieved at all investigated wavelengths. The size of the ablated particles ranged from a few hundred nanometres to a few micrometres. A quantitative analysis was performed using the QuPath-0.3.1 software. The evaluation was challenging because the particles clustered into piles and overlapped during the drying of the droplets. Therefore, particle boundaries were determined individually in the SEM images. Around 10 mm^2^ image area was analysed in case each sample. As an approximation, after determining the area covered by each particle, circles of equal area were assigned to the particles outlined in the images, and the diameter of each circle was used as the characteristic parameter of the particle size when plotting the size distribution diagram (Fig. 8).

### Fast photography studies

As previously proposed^12^, the production of meloxicam particles by pulsed laser ablation in liquid can be described by the following step sequence: 1. the pulse energy is absorbed in a small volume of the surface layer of the target; 2. there is an intense and rapid rise in temperature, and the meloxicam evaporates and decomposes in the uppermost volume, resulting in an explosive gas release and plasma expansion; 3. recoil forces emerge, which tear off partly molten and solid particles from the remaining surface. If the particles accumulate in the liquid medium, there is a possibility of “secondary” fragmentation, when the already dispersed particles are irradiated by the laser pulses. In our case this effect could not be significant due to the low particle concentration and also the fluence reaches the ablation threshold only close to the focus.

The time period between 50 ns and 2 ms after the laser pulse impact was investigated using λ = 532 nm pump pulses and direct probe illumination. To minimize the glow of the plasma, we tried to find the lowest possible fluence at which the formation of particles can already be observed.

**Figure 9:**
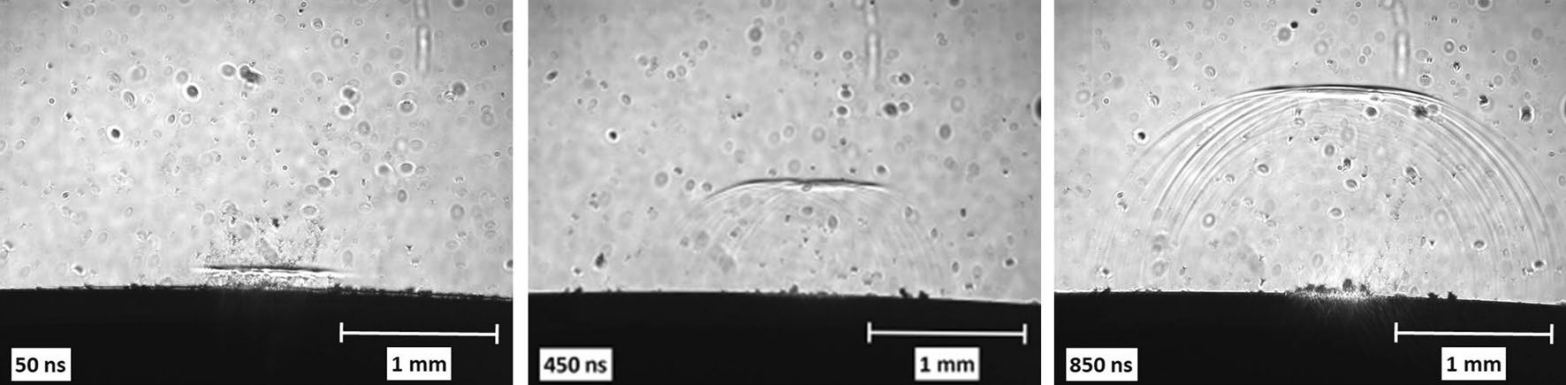
Fast photography images of pulsed laser ablation (λ = 532 nm, F = 3 J/cm^2^) of meloxicam in distilled water.

For demonstration purposes, at 3 J/cm^2^ only the wave front induced by the expanding plasma was visible, and there was no material ejection (Fig. 9).

**Figure 10:**
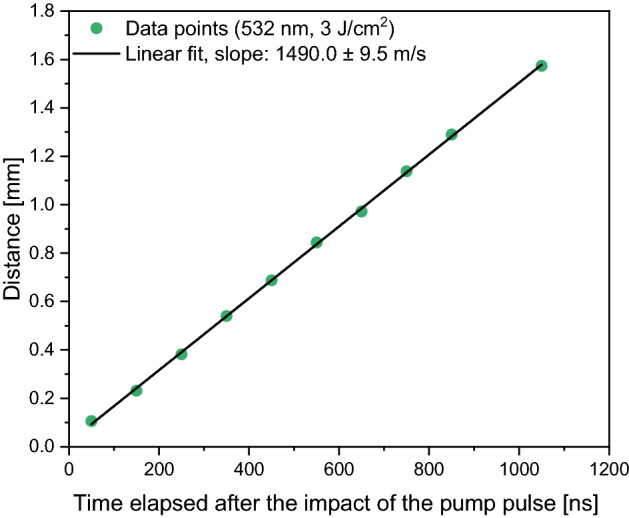
Distance of the wave front from the pastille surface as a function of time. The data points are from the fast photography measurements (λ = 532 nm, F = 3 J/cm^2^).

After evaluating the images with ImageJ, we plotted the distance of the wave front from the surface over time (Fig. 10) and fitted the data points with a linear function. For the slope we obtained v = 1490.0 ± 9.5 m/s, which is only slightly above the speed of sound propagating in water: v_w_ = 1480 m/s (T = 20 °C), indicating that the observed waves were rather acoustic than shock waves. This means that even if there were initial shock waves induced by the expanding plasma, they diminished quickly.

We tried to increase the lifetime of the shock wave by raising the fluence to 6 J/cm^2^, but still no shock wave was detected. On the other hand, we witnessed intense material ejection.

**Figure 11:**
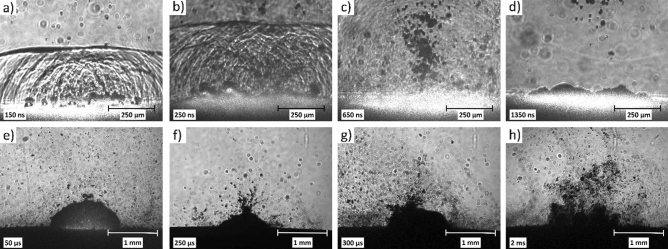
Fast photography images of PLAL (λ = 532 nm, F = 6 J/cm^2^) of meloxicam in distilled water. **(a)–(c)** Formation and propagation of the wave front; **(d)–(g)** Rebounding dynamics of the cavitation bubble with increasing particle ejection; **(h)** Disintegration of the cavitation bubble and particle release/dispersion in the water.

We can distinguish three main stages in the process of pulsed laser ablation in water, which can be clearly seen in the fast photography images (Fig. 11). In the first few hundred nanoseconds, a wave front propagates in the medium (Fig. 11a-c). The main front is the sum of numerous small waves originating from various point-like sources on the target surface. The interference of these waves and their scattering on some ejected particles can also be seen. In addition, changes in the reflective index of the medium due to an increase in temperature could also contribute to the fringes behind the main wave front.

The second stage lasts from a few microseconds to a few hundred microseconds and involves the formation and size changes of the cavitation bubble (Fig. 11d–g). This repeated rebounding of the cavitation bubble has been described previously in the framework of bubble dynamics for the formation of metal (copper) nanoparticles by laser ablation in liquid^26^. The generated particles are mainly inside the cavitation bubble, but as the bubble pulsates, more and more particles are ejected. Small gas bubbles appear and rise to the liquid surface alone or sometimes with attached particles. Contrary to a previous publication^27^, the presence of persistent gas bubbles is not typical in our setup, perhaps due to the smaller applied ablation frequency.

Finally, in the third stage, after a few milliseconds, the cavitation bubble breaks up into smaller bubbles that rise to the surface of the water and all the particles trapped in the cavitation bubble are dispersed in the water (Fig. 11h). Similar mechanism was presented for various targets and liquids^28–30^.

We optimized the setup and the settings to be able to detect initial shock waves. From the optical fibre, the probe pulse was led to the Michelson interferometer providing two probe pulses with a fixed time separation. This arrangement enabled us to estimate the propagation velocity more accurately. To avoid any overlap of the pump and probe laser pulses and to minimize the interfering scattered light, the λ = 1064 nm laser beam was applied as the pump. We increased the fluence until the shock waves appeared. The ablation rapidly degraded the surface, so we had to move the target between consecutive ablating shots to provide a fresh surface. Additionally, the jitter of the timing, the porosity of the target and the slight variance of the laser pulse energy all contributed to a certain degree of uncertainty in the quantitative analysis.

**Figure 12:**
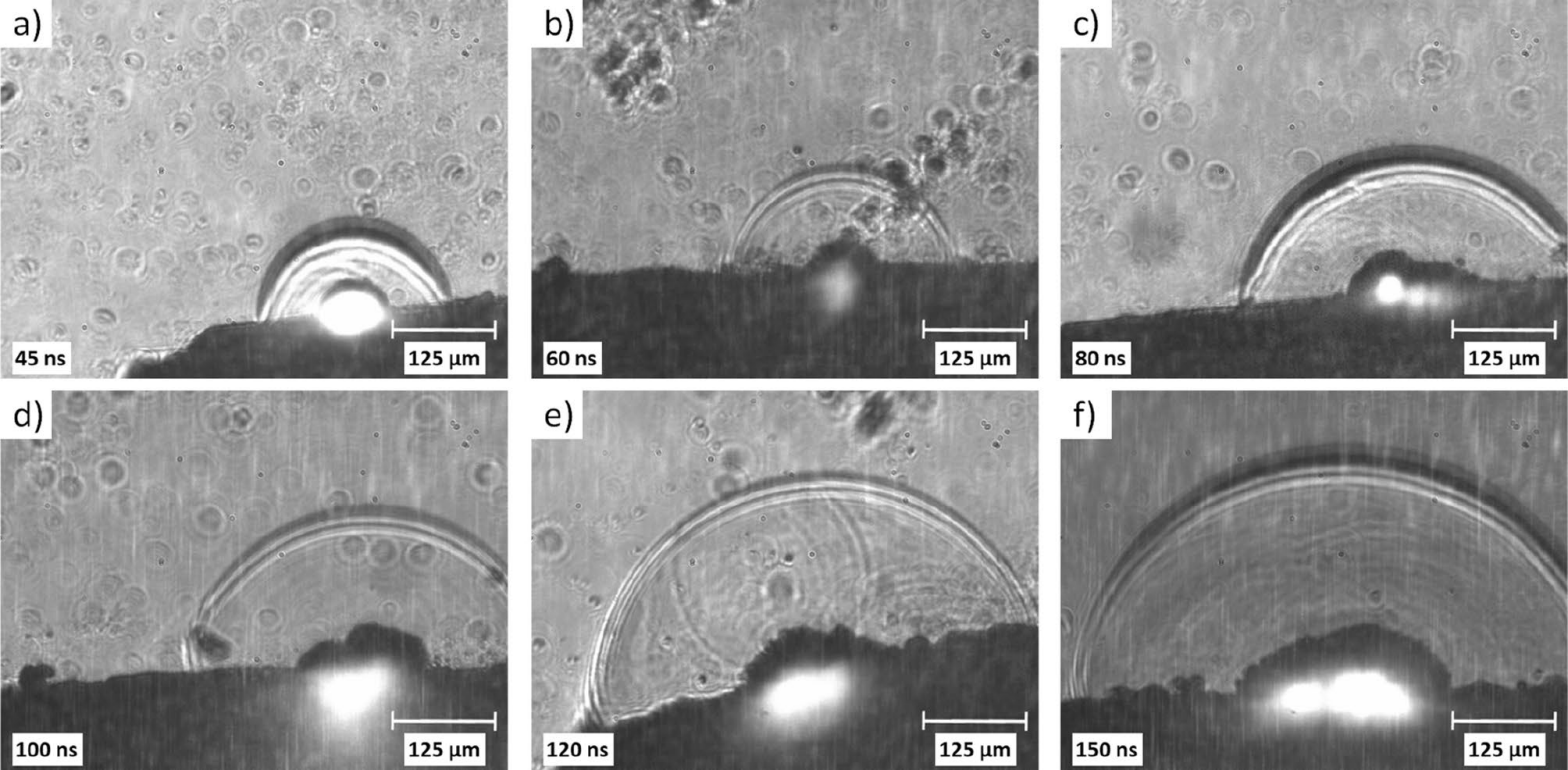
Fast photography images of pulsed laser ablation (λ = 1064 nm, F = 18 J/cm^2^) of meloxicam in distilled water. **(a)–(f)** Formation and propagation of the wave front double-exposed at two moments with *t* = 8 ns time separation; **(c)–(f)** Generation and size changes of the cavitation bubble.

Figure 12 presents pictures taken by using this optimized setup and capturing the initial phases of shock wave formation and the appearance of the cavitation bubble. Over time, due to the specific imaging scheme, it appears as if two wave fronts are propagating with decreasing spatial separation. The cavitation bubble emerges and grows.

**Figure 13:**
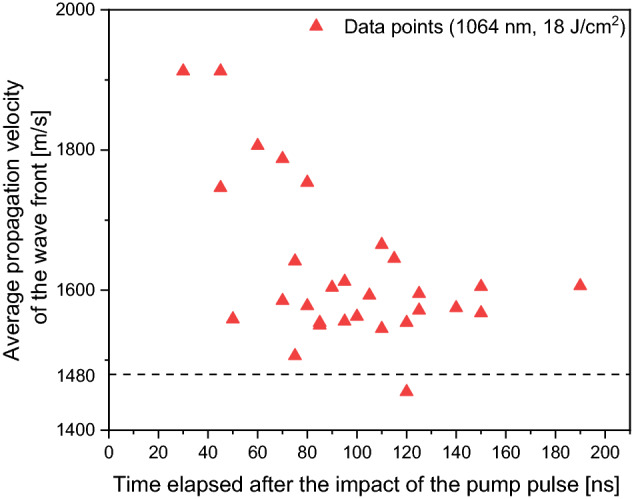
Time dependence of the propagation velocity of the wave front produced by PLAL (λ = 1064 nm, F = 18 J/cm^2^) of meloxicam in distilled water.

Using ImageJ, the spatial separation/distance (x) of the wave fronts was measured on each image. The Michelson interferometer introduced a t = 8 ns time separation between the two probe pulses. At a given moment after the impact of the pump pulse, by dividing the current distance by the fixed time separation, we could calculate the average propagation velocity of the wave front between the two moments recorded with the probe pulses (Fig. 13). The data points show that the average propagation velocity goes beyond the velocity of sound in water (v_w_) and then follows a decreasing trend over time, gradually approaching v_w_ = 1480 m/s. As the shock wave left the field of view, we could not get any velocity data after 200 ns delay where the saturation seems to be around 1550 m/s (Fig. 13). However, on the basis of the wave front propagation measurements presented in Fig. 10, we can assume that in the end the saturation will be around v_w_ = 1480 m/s. In conclusion, the initial shock wave becomes an acoustic wave within a few tens or hundreds of nanosecond, depending on the applied fluence.

### Investigating pharmaceutical applicability (solubility, cytotoxicity, anti-inflammatory effect)

**Figure 14:**
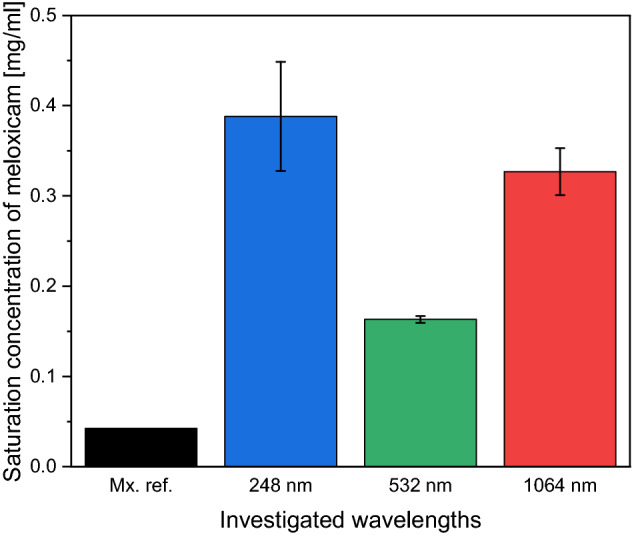
Solubility data of the commercially available meloxicam (Mx. ref.) and the samples produced with different ablation wavelengths (248 nm, 532 nm, 1064 nm).

Solubility studies showed that higher amount of meloxicam could be dissolved in buffer solution from the particles prepared with the PLAL method than from the original meloxicam powder in case of all applied laser wavelengths (Fig. 14).

Compared to the saturation concentration of the reference meloxicam (0.043 mg/ml), a nine-, four- and eightfold increase of saturation concentration was measured when meloxicam particles produced by PLAL at 248 nm, 532 nm and 1064 nm were dissolved. Quantitatively, this resulted in saturation concentrations of 0.388 ± 0.0605 mg/ml (for λ = 284 nm), 0.163 ± 0.004 mg/ml (for λ = 532 nm) and 0.327 ± 0.026 mg/ml (for λ = 1064 nm).

**Figure 15:**
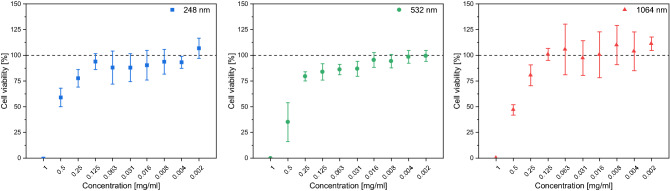
Cell viability in case of the investigated ablation wavelengths (248 nm, 532 nm, 1064 nm).

The non-toxic limit was defined as the highest concentration which still ensures 100% cell viability. The therapeutic dose of meloxicam in marketed medicines is 7.5 or 15.0 mg *per os* and 1/10 part of this dose is recommended for pulmonary delivery ^31,32^, therefore 1 mg/ml concentration was selected as a maximum concentration for the cytotoxicity studies. The reference meloxicam showed no toxicity at concentrations below 0.125 mg/ml. Cytotoxicity measurements showed that the samples generated by PLAL at λ = 248 nm and 1064 nm had no measurable cytotoxic effect below 0.125 mg/ml, while in case of λ = 532 nm, the non-toxic limit was around 0.016 mg/ml (Fig. 15). The samples produced by PLAL at λ = 248 nm and 1064 nm were in the same cytotoxicity range as the reference meloxicam, while the λ = 532 nm generated compound was the most cytotoxic. These determined non-toxic concentrations were used in the anti-inflammatory measurements.

**Figure 16:**
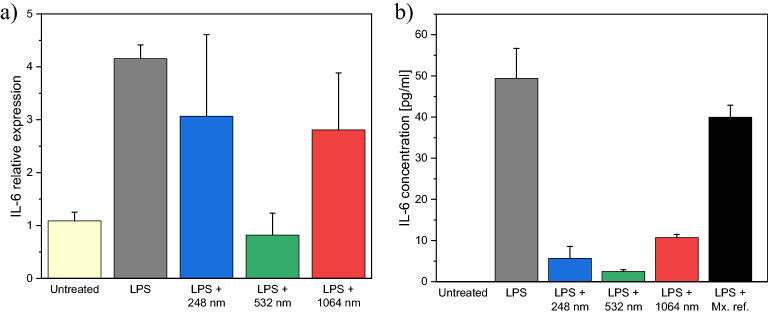
**(a)** Relative expression and **(b)** concentration of IL-6 in case of untreated A549 cells (Untreated), LPS-treated A549 cells (LPS), A549 cells treated with LPS and meloxicam suspension generated by laser ablation at respective wavelengths (LPS + 248 nm, LPS + 532 nm and LPS + 1064 nm). In case of the IL-6 concentration (**b**), the data of A549 cells treated with LPS and reference meloxicam suspension (LPS + Mx. ref.) is also presented.

To test the anti-inflammatory effect, LPS, a strong proinflammatory agent was utilized to increase the IL-6 production of A549 cells^33^. By measuring the IL-6 content after treatment with the PLAL produced drug samples, we could describe and compare their anti-inflammatory effects. The added LPS significantly elevated the IL-6 relative expression compared to the untreated group. The IL-6 relative expression quadrupled in case of LPS-treated cells (4.2 ± 0.2) compared to the untreated cells (1.1 ± 0.2). By comparing the relative expression of IL-6 in cells treated with LPS + drug and cells treated with LPS only, we found that a moderate decrease (∼25%) could be achieved by adding the UV (λ = 248 nm) and IR (λ = 1064 nm) laser ablation produced samples, and a significant reduction (∼75%) by adding the VIS (λ = 532 nm) laser ablated sample (Fig. 16a). Quantitatively, this resulted in IL-6 relative expressions of 3.1 ± 1.5 for UV, 0.8 ± 0.4 for VIS and 2.8 ± 1.1 for IR.

To determine whether these relative expression levels correlated with the protein levels, ELISA measurements were conducted to measure the IL-6 concentrations in the supernatants (Fig. 16b). In the untreated group there was no measurable IL-6 level. Compared to the untreated cells (0.0 ± 0.0 pg/ml), the IL-6 concentration increased significantly as a result of LPS-treatment (to 49.4 ± 7.3 pg/ml). When reference meloxicam was added to the LPS treated cells, the IL-6 concentration decreased by 20% (to 39.9 ± 2.9 pg/ml) compared to the cells treated with LPS only. After LPS-treatment, the addition of laser ablated samples considerably reduced the IL-6 concentrations: by 89% (to 5.6 ± 2.9 pg/ml), 95% (to 2.5 ± 0.4 pg/ml) and 78% (to 10.7 ± 0.8 pg/ml) using UV, VIS and IR lasers, respectively. Based on the decrease in IL-6 cytokine concentration, all PLAL produced samples showed a significant anti-inflammatory effect, much stronger than the reference meloxicam. According to the measurements, the λ = 532 nm generated sample had the best anti-inflammatory properties despite the lowest dose required based on the cytotoxicity measurements.

## Conclusions

Pulsed laser ablation of meloxicam in distilled water has proved to be an effective and chemical free particle size reduction technique at all three investigated wavelengths.

The FTIR, Raman spectroscopy and HPLC–MS studies showed that chemical decomposition was negligible during ablation. Meloxicam impurity B was present in a relatively small quantity. The particles kept their crystallinity throughout the process and their morphology resembled that of broken up crystals. The initial ~ 30 µm size of the meloxicam particles could be reduced to the size range between a few hundred nanometres and a few micrometres, optimal for pulmonary delivery. Fast photography analysis confirmed that mechanical shredding is the main process leading to particle size reduction. Pharmaceutical applicability is supported by the significant (nine-, four- and eightfold) increase in solubility of the ablated samples at the respective ablation wavelengths. Despite the similar (∼0.125 mg/ml at λ = 248 and 1064 nm) or greater (∼0.016 mg/ml at λ = 532 nm) cytotoxic effect of the PLAL produced suspensions, the anti-inflammatory effect was excellent (IL-6 concentration decreased by 89, 95, 78% at λ = 248, 532, 1064 nm, respectively) and highly exceeded that of the reference meloxicam.

Based on the solubility, cytotoxicity and anti-inflammatory properties obtained, we expect that the same therapeutical effect can be achieved with a significantly lower drug dose by using particles shredded by the PLAL technique.

Therefore, meloxicam particles generated by pulsed laser ablation in distilled water could be suitable for *per os* or pulmonary administration. Alternatively, meloxicam suspended in water could be transformed to solid intermediate product for preparation of different dosage forms (tablets, capsules or Dry Powder Inhalers, etc.).

## Data Availability

The datasets generated during and/or analysed during the current study are available from the corresponding author on reasonable request.
